# DNAJs: more than substrate delivery to HSPA

**DOI:** 10.3389/fmolb.2015.00035

**Published:** 2015-06-30

**Authors:** Suzanne L. Dekker, Harm H. Kampinga, Steven Bergink

**Affiliations:** Department of Cell Biology, University Medical Center Groningen, University of GroningenGroningen, Netherlands

**Keywords:** DNAJ, ubiquitin E3 ligases, HSP70 heat-shock proteins, degradation, protein folding

## Abstract

Proteins are essential components of cellular life, as building blocks, but also to guide and execute all cellular processes. Proteins require a three-dimensional folding, which is constantly being challenged by their environment. Challenges including elevated temperatures or redox changes can alter this fold and result in misfolding of proteins or even aggregation. Cells are equipped with several pathways that can deal with protein stress. Together, these pathways are referred to as the protein quality control network. The network comprises degradation and (re)folding pathways that are intertwined due to the sharing of components and by the overlap in affinity for substrates. Here, we will give examples of this sharing and intertwinement of protein degradation and protein folding and discuss how the fate of a substrate is determined. We will focus on the ubiquitylation of substrates and the role of Hsp70 co-chaperones of the DNAJ class in this process.

## Protein stress

The capacity of the protein quality control network is limited and collapse occurs when the system is overwhelmed. This can occur after sudden massive stress including temperature shifts, nutrient deprivation or pathogenic infection (Lee, 1992; Scheuner et al., 2001; Kaufman et al., 2002; Schelhaas et al., 2007; Morimoto, 2008). These types of stresses are usually sensed and the organism responds with the activation of stress pathways, such as the heat shock response after temperature elevation (Morimoto, 2008).

Also “normal” conditions in the cell, for example the increased synthesis of proteins during cell cycle progression (Morimoto, 2008) can be considered as physiological protein stress requiring increased folding capacity and adaptations in the quality control network. The difference between these two types of protein stress is that the latter is enlisted and that substrate recognition and fate are pre-determined, for example cyclins are degraded upon orchestrated phosphorylation events (Murray, 2004). In contrast, sudden or accidental protein stress is unpredictable and requires a system that can recognize the client(s), which lack(s) a common motif that is usually present in the regular enlisted clients. Typically, these accidental clients become unfolded after stress or are intrinsically misfolded (i.e., genetically encoded).

## Protein stress and its impact on human pathology

Protein stress can accumulate over time as well, as is the case in many diseases that are associated with an accumulation of misfolded proteins. Often this type of slowly accumulating protein damage is—initially—not triggering a strong stress signaling, but remains undetected. Yet, this type of stress may also overwhelm the protein quality control network and cause a similar collapse, ultimately resulting in the associated pathology. Diseases driven by protein stress range from cataract, type II diabetes, atrial amyloidosis to neurodegenerative diseases (Chiti and Dobson, 2006).

Moreover, it is known that hypomorphic mutations, a partial loss of gene function, can lead to an accelerated phenotype in the background of a compromised protein quality control network (Ben-Zvi et al., 2009). Suggesting that genome alterations can lead to metastable or aberrant polypeptides that stochastically accumulate in time. If true, the source of protein stress would expand to single nucleotide polymorphisms (SNPs) and somatic mutations that slowly accumulate as we age.

## Molecular chaperones

Heat Shock Proteins (HSPs) play a central role in protein homeostasis and are upregulated by the diverse protein stress signaling pathways in cells under many conditions, most prominently acute stress, that challenge protein homeostasis (Morimoto, 2008). For various HSPs, it has been established that they have so-called molecular chaperone activity. Usually, this is associated with folding nascent chains into their native state or refolding of stress-unfolded proteins. However, their activities are also needed for degradation of misfolded or mutant proteins and even for remodeling of active protein complexes. As a matter of fact, the most ancient members of the molecular chaperones, DnaJ, and DnaK (Hsp70/HSPA), were originally identified as essential components controlling (dis)assembly of phage lambda replication complexes (Konieczny and Zylicz, 1999).

**Figure 1:**
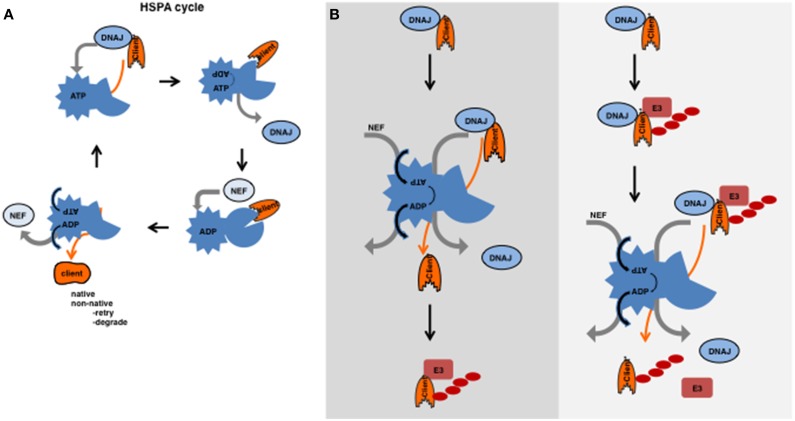
**(A)** HSPA cycle. HSPAs (HSP70s and HSC70s) contain an N-terminal ATPase domain connected by a hydrophobic linker to a variable C-terminal peptide-binding domain (Hartl et al., 2011). This peptide-binding domain binds to a stretch of hydrophobic residues flanked by positive residues, which is predicted to occur every 40 amino acids (Frydman et al., 1999). In the folded state, these hydrophobic residues are buried inside, but are exposed in the unfolded or misfolded state. The energy obtained from the hydrolysis of ATP is required for assisted folding. However, the exact mechanism how an HSPA supports folding is still not yet completely understood. HSPA functions with the help of co-chaperones that orchestrate the cycle of ATP hydrolysis and substrate/client binding and release. DNAJs recognize clients and subsequently bind to the ATP-bound form of HSPA; upon binding of the DNAJ-client complex, ATP is hydrolysed by the HSPA and the DNAJ is released. Upon hydrolysis and DNAJ release, the peptide-binding-domain of HSPA undergoes a conformational change and clams around the polypeptide (the substrate) (Jiang et al., 2007; Swain et al., 2007; Bertelsen et al., 2009). The NEF has affinity to the ADP-bound form and mediates the exchange of ADP for ATP. The client has less affinity for the ATP-bound form of HSPA and releases together with the NEF. As a result, the client can fold or will re-enter the cycle if not completely folded, or somehow can be transferred to degradation machineries. **(B)** Substrates ubiquitylated before or after the action of HSPA. On the left, the canonical model is depicted in which an intrinsically unstable substrate is first recognized by a DNAJ then transferred to the HSPA cycle and after a futile folding event ubiquitylated and subsequently degraded. On the right, a model is presented in which an unfolded substrate is recognized by a DNAJ after which an E3 ligase can ubiquitylate it and the HSPA cycle acts on the ubiquitylated substrate. After the action of HSPA, the substrate is liberated from both the DNAJ and the E3 ligase (complex) and targeted for degradation.

A wide network of molecular chaperones exists that encompasses the chaperonins, the HSPAs (Hsp70s and Hsc70s, eleven in humans) with its co-chaperones: the DNAJs (50 members in humans) and the Nucleotide Exchange factors (NEF, 13 members in humans), the HSPBs (small HSPs, 10 members in humans) and the HSPCs (Hsp90s, five members in humans) (Kampinga and Craig, 2010). Here we will focus on the DNAJs in the HSPA system (Figure 1A), as these are involved in substrate recognition and seem to function at the crossroad of folding and degradation.

## The DNAJs: a crucial role in protein quality control

The HSPAs function with the help of several co-chaperones and collaborate with the other chaperones. The most important co-chaperones are the DNAJs and the NEFs. DNAJs recognize substrates/clients and subsequently bind to the ATP-bound form of HSPA (for the HSPA cycle see Figure 1A).

DNAJs are defined by the presence of a J-domain, an approximately 70 amino acid domain that consist of four alpha helices and an accessible loop to which the (ATP-bound) ATPase domain of HSPA binds (Jiang et al., 2007). In this loop lies a Histidine Proline Aspartate (HPD) motif, which is crucial for binding to HSPAs. DNAJs are divided based on their domain structure into three classes, DNAJAs, DNAJBs and DNAJCs (Kampinga and Craig, 2010). Briefly, in both DNAJAs and DNAJBs, the N-terminal J domain is followed by a Glycine/Phenylalanine-rich stretch. In the DNAJA class proteins, two domains (CTDI and CTDII) are located at the C-terminus that are involved in substrate recognition by forming a hydrophobic pocket that binds to the substrate (Li et al., 2003). The first of these CTDs contains a zinc finger. The C terminus in the DNAJB lacks the organization into two CTDs or and more specifically contains no zinc finger and specific information of substrate binding regions is still lacking. The DNAJC class comprises any J-domain containing protein that does not fit in the A or B class.

A wide variety of domains can be found in the different J proteins. Many of these are known or thought to be involved in substrate interactions, thus a division based on substrate recognition has been proposed as well (Kampinga and Craig, 2010). Many J-proteins are believed to have a promiscuous client binding (mostly from class A and B and a few from class C) while others seem to display high substrate specificity (mostly class C).

## Interplay between degradation and folding

The notion that there are around 50 DNAJs, 11 HSPAs, and 13 NEFs at least suggest a high potential of many possible combinations, theoretically resulting in flexibility and a broad substrate range. Some DNAJs and HSPAs are localized to specific organelles and seem to form combinations with the compartment specific Hsp70 (HSPA) members, but for other (cytosolic) HSPAs it has been shown that they can indeed operate with different DNAJs (Kampinga and Craig, 2010). The interplay between the HSPA cycle and the degradation machineries makes matters even more complex. The idea that the fate of a protein is determined by this interplay of the chaperone systems and the degradation systems is often referred to as the triage decision (Connell et al., 2001; Houck et al., 2012). The classical view is that the cell first attempts to (re)fold the damaged or metastable protein. If folding fails, the cell will then attempt to degrade it (Figure 1B). Many proteins that are part of the folding machinery interact with the cellular degradation machinery to make this transition smoothly. For example, DNAJB2 interacts with ubiquitylated proteins via its ubiquitin interacting motifs (UIM) (Westhoff et al., 2005) and Bag1 (a NEF) contains a ubiquitin-like (UBL) domain which are known to interact with subunits of the proteasome (Lüders et al., 2000). An alternative view would be that degradation and (re)folding act in parallel and that chaperones are an integral part of the degradation machinery and have a different function besides folding. These scenarios are not mutually exclusive and, depending on the substrate, both might happen.

How the HSPA machinery selects and recognizes the different types of substrates and which parameters determine the fate of the clients (folding, degradation or aggregation) are still open questions. Here we will give examples of clients that are dealt with by different combinations of DNAJs and HSPAs. Moreover, we will discuss the recent insights in the interplay between the HSPA cycle and the ubiquitin proteasome system (UPS) (Box 1).

Box 1 **Protein Degradation.**

The various proteases in the cell are capable of degrading targeted substrates. As a general theme, access to proteases is restricted either due to a high specificity for specific substrates, a common but inducible recognition signal (e.g., the proteasome), or spacious confinement (e.g., the lysosome). The UPS and autophagy are the systems that degrade the bulk of proteins.

**UPS**

Recognition by the 26S proteasome is done by lysine (K) 48-linked polyubiquitin chains conjugated to substrates that require degradation. Ubiquitin is a polypeptide that is conjugated to target proteins by the subsequent action of three enzymes, an E1 (activating enzyme), an E2 (conjugating enzyme) and an E3 (ligating enzyme). The ligases are responsible for substrate recognition and thus determine the specificity, whereas the E2s usually mediate the conjugation of ubiquitin to the substrate. Occasionally an E4 ligase is required to extend already existing mono- or oligo-ubiquitylation events to polyubiquitin trees. The C-terminal glycine of ubiquitin either forms a peptide bond with the N-terminus of a substrate or an isopeptide bond with the epsilon-N in internal lysines of the substrate (although conjugation to cysteine and serine have been reported as well). Due to the internal lysines in ubiquitin itself a so-called polyubiquitin tree is build. Polyubiquitylation with a K48 linkage is considered canonical for degradation. Indeed, monoubiquitylation or other types of polyubiquitin trees with different linkage are not necessarily recognized as targeted for destruction. Receptors at the 26S proteasome recognize the polyubiquitin trees, which are cleaved off and recycled. The 26S proteasome is a multimeric complex that consists of a 20S and 19S component. After deubiquitylation, the substrate is unfolded by ATPases associated with various cellular activities (AAA-ATPases) of the 19S, and funneled into the proteolytic chamber (20S). Only when a protein resides in this chamber it is cleaved into peptides.

**Autophagy**

Autophagy is a process in which a double membrane-vesicle (the autophagosome) containing the cargo is targeted to, and fused with the lysosome in which the degradation (hydrolysis) takes place. The cargo consists of proteins, protein-complexes, aggregated proteins and even entire organelles. Autophagy is mediated by a set of proteins and includes two types of ubiquitin-like conjugation reactions; the most crucial one is the conjugation of LC3 or ATG8 (in yeast) to phosphoatidylethanolamine at the expanding autophagic membrane. Originally it was thought that autophagy is mostly nonspecific and just randomly engulfs cytoplasm. However, the current dogma is that most autophagy is in fact specific. Again, ubiquitylation can form the signal that is recognized by specific receptors that both bind polyubiquitylated substrates and LC3, the double membrane then encloses the cargo and translocates to the lysosome.

## *De novo* protein folding

The folding of newly synthesized proteins is different from refolding reactions, partly due to the vectorial nature of translation. Indeed, early studies clearly demonstrated that nascent chain folding and refolding require different sets of chaperones (Frydman et al., 1994, 1999). Folding of newly synthesized proteins can occur co-translationally and sometimes also post-translationally. In prokaryotes, folding involves many different partners including trigger factor which prevents misfolding of nascent chains by delaying protein folding (Agashe et al., 2004). Although far from completely resolved, in co-translational folding, the different domains fold separately. As often, the fundamental studies in yeast have shed light on this process (Albanèse et al., 2006). Two complexes are associated with the ribosome and the emerging polypeptide. First, the nascent polypeptide associated complex (NAC), a heterodimer that consists of an alpha and a beta subunit, binds to all nascent chains. Second, a set of Hsp70s (Ssb1 and 2) is bound to translating ribosomes, which function together with the DNAJ Zuo1 and the NEF Sse1 and bind to the polypeptide that extrudes from the ribosomal exit tunnel. Zuo1 together with Ssz1 (another Hsp70) forms the Ribosome Associated Complex (RAC), and is thought to recruit Ssb1 and 2 to the ribosome. Global identification of Ssb1/2 substrates revealed that especially the more difficult substrates (e.g., longer, several domains, beta sheet enriched, and aggregation prone) are clients (Willmund et al., 2013). Beside Zuo1, two other DNAJ proteins have been identified as being important for nascent chain folding namely Ydj1 and Sis1. These DNAJs function with the “soluble” or cytoplasmic Hsp70s (the Ssa1-4 proteins) and the chaperonin system to guide the final (post-translational) folding of newly synthesized polypeptides (Kim et al., 2013).

Most of these yeast proteins have mammalian counterparts: Ssz1 is homologous to HSPA14, Zuo1 is homologous to DNAJC2 [also called, Mpp11 (M-phase phosphoprotein 11) (Hundley et al., 2005) or ZRF1 (zuotin related factor 1) (Richly et al., 2010)], Ydj1 is homologous to DNAJA1 and Sis1 is homologous to DNAJB1. HSPA8 is associated with the ribosome and homologous to Ssb2 (Hundley et al., 2005).

## Quality control at the ribosome

**Figure 2:**
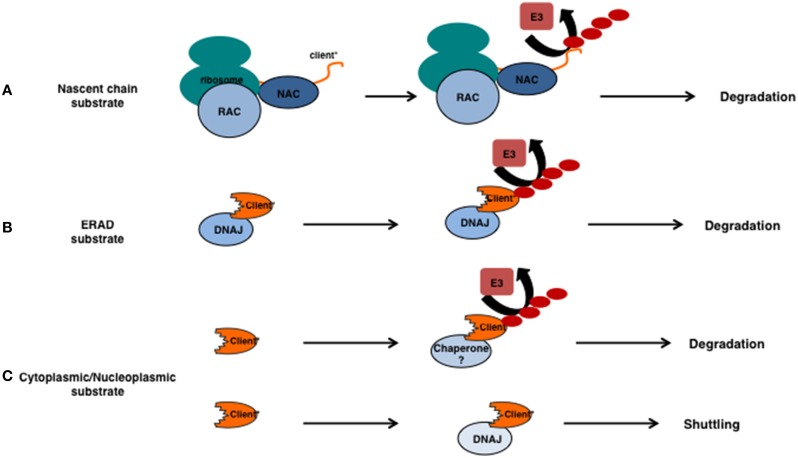
**Involvement for chaperones in substrate ubiquitylation. (A)** The relationship between chaperones and ubiquitin E3 ligases for unstable nascent chains. Unstable nascent chains are bound by the chaperone systems present at the ribosome (here the NAC and RAC) and ubiquitylation of the emerging polypeptide happens co-translationally. **(B)** ERAD substrates that are intrinsically unstable (indicated by the ^*^) are recognized by DNAJs prior their ubiquitylation and retrotranslocation. **(C)** If cytoplasmic or nucleoplasmic substrates require chaperones prior their ubiquitylation is currently unclear. Sis1/DNAJB1 is required for the nuclear relocalization of certain unstable substrates in an ubiquitin-independent manner.

Translational errors may occur due to aberrant mRNAs, misfolding due to intrinsic properties of the nascent chain itself or to other events that lead to stalling of the translational machinery. As a general theme, these errors are sensed during translation and the emerging polypeptide is co-translationally ubiquitylated, released and degraded. Depending on the type of translational problem and substrate, different chaperones and components of the UPS are involved. For example, long stretches of basic amino acids are recognized by the NAC complex and ubiquitylated by the E3 ligase Not4 (Dimitrova et al., 2009). Other types of translational difficulties, such as the translation of the polyA sequence that leads to pausing, require another E3 ligase, namely Ltn1. The ubiquitylated polypeptide requires Cdc48^Ufd1/Npl4^ (p97/VCP in mammalians), a ubiquitin/ubiquitin-like-modifier-specific segregase (Jentsch and Rumpf, 2007), to remove it from the ribosome (Brandman et al., 2012; Defenouillère et al., 2013; Verma et al., 2013). Thus, translational errors usually result in immediate, co-translational, degradation (Figure 2A) in which chaperones act before ubiquitylation.

Degradation of the emerging polypeptide can in principle always occur at the ribosome, even in the absence of translational errors, as shown by elegant experiments using tandem expressed degradation-reporters (Turner and Varshavsky, 2000). This implies that folding or degradation is an intrinsic fate of the client rather than it is driven by different sets of chaperone machines. In other words, the different ribosome-associated chaperone complexes (in yeast Ssb1/2, RAC, and NAC) prevent aggregation during translation which -depending on the substrate- can result in folding or maintain the substrates/clients accessible to the various E3 ligases that are either present at, or have access to the ribosomes (examples in yeast: Ltn1, Not4, Ubr1).

## Chaperones: the prelude for ubiquitylation of proteasomal clients?

The idea that unfolded or misfolded clients, similar to nascent chains, need to be kept accessible/soluble in order for a protein quality control E3 ligase to work seems appealing. The idea would be that a chaperone binds first to a potential substrate and keeps this substrate soluble or accessible for the E3 ligase to ubiquitylate this target. The requirement for a chaperone as a recognition or accessibility factor for ubiquitin E3 ligases would not be necessary for regular or enlisted clients (e.g., the cyclins), but only for those that become (partly) unfolded by a random stress event or for those clients that are intrinsically (genetically encoded) misfolded. Chaperones of the HSPB and DNAJ class are well known to be able to recognize these clients and thereby ensure their solubility. In the canonical view, these clients are next transferred to the HSPAs (Hsp70s). Release from the HSPAs occurs via NEFs and interactions with E3 ligases at which level client-fate is determined. However, as we will explain below, evidence is accumulating that other scenarios may also be operational (Figures 1B, 2).

## Ubiquitylation and DNAJs in ERAD

Clear examples where a DNAJ precedes the action of an E3 can be found within ER associated degradation (ERAD). Proteins that are targeted to the ER, but are unwanted because they are intrinsically unstable, aggregation prone or unfolded, are cleared via the ERAD pathway. For ER luminal and transmembrane clients, DNAJs (DNAJB11, DNAJB9, and DNAJC10 also known as Erdj3, -4, and -5), recognize misfolded clients that subsequently are ubiquitylated by the membrane embedded E3 ligases Hrd1 or RMA1 and the E4 ligase gp78 (Buchberger et al., 2010). For those clients that expose their unstable region to the cytosol, soluble-DNAJs play a role in conjunction with the E3 ligase Doa10 (Ravid et al., 2006). After or during ubiquitylation, all ERAD clients require retro-translocation over, or dislocation from the lipid bilayer, a process that is mediated by the p97/Cdc48^Ufd1/Npl4^ complex (Tsai et al., 2002).

Also for integrated membrane proteins, DNAJs may act prior to E3 ligases in degradation. Several model substrates exist exposing their unstable region to the cytosol and for some of these the relationship between chaperone function and ubiquitylation status has been determined. For example degradation and ubiquitylation of the Doa10 substrates Ste6^*^ and PMA1^*^ depends on the cytosolic Hsp70s (Ssa1-4) and the (cytosolic) DNAJs Ydj1 and Hlj1 (Han et al., 2007; Nakatsukasa et al., 2008) (Figure 2B). Other DNAJs are also involved in Doa10-mediated substrate ubiquitylation as Sis1, but not the cytosolic Hsp70s (Ssa1-4), is required to ubiquitylate the degradation prone part from the kinetochore protein Ndc10. Instead, Hsp70s are required to prevent aggregation of the ubiquitylated client (Shiber et al., 2013) and clearly function after ubiquitylation. These results are in line with the idea that E3 ligases involved in clearing misfolded substrates require a molecular chaperone for their functioning (Figure 2).

However, for other substrates (e.g., soluble targets of the protein quality control E3 ligases Ubr1 and San1) Sis1 (or Ydj1) is not a prerequisite for ubiquitylation but is essential for efficient client degradation (Park et al., 2013; Mehnert et al., 2015; Miller et al., 2015) and thus seem to play a role downstream of ubiquitylation.

## Ubiquitylation and DNAJs of cytoplasmic and nucleoplasmic clients

Whereas, translation quality control and ERAD are vectorial by nature, the organization of quality control over soluble proteins is not. If the soluble (stress induced or accidentally misfolded) clients in the cytosol and nucleoplasm also require a chaperone prior ubiquitylation is currently unclear. The notion that overexpression of certain DNAJs accelerates degradation of substrates is suggestive of a direct impact of DNAJs on degradation/ubiquitylation (Westhoff et al., 2005). However, clear evidence that DNAJs or HSPBs are required for ubiquitylation of these accidental clients is currently lacking (Figure 2C). Deletion of individual DNAJs or other molecular chaperones typically does not result in accumulation of their unmodified clients. It is not unlikely that there is functional redundancy between the different and numerous chaperones (e.g., DNAJs) in these compartments making the fate of substrates/clients harder to study. Moreover, to distinguish this process from translational protein quality control (Wang et al., 2013), a substrate that becomes unstable after translation vs. a constitutively unstable substrate, is required. For example a substrate that after the addition of a specific small molecule triggers unfolding of that substrate would be a great asset. This type of substrate would enable the study of post-translational protein quality control under normal, non-stressed, conditions. This type of research is especially important as, in many of the aforementioned diseases, protein stress occurs post-translationally.

## Nuclear transport of cytosolic substrates

As mentioned above, ERAD substrates are degraded in the cytoplasm and retro-translocation is necessary as the proteasome is not present in the ER. One might suspect that degradation of cytoplasmic clients is more straightforward as the proteasome is present in the same compartment. However, recent evidence suggests that, at least for some substrates, this is not the case. Instead, cytosolic model substrates were found to shuttle to the nucleus for degradation (Figure 2C) (Heck et al., 2010; Prasad et al., 2010; Park et al., 2013). This transfer across the nuclear envelope depends on the DNAJ Sis1 (DNAJB1 in humans) (Park et al., 2013). Increasing the proteotoxic stress hampers this process, probably due to a sequestration of Sis1 to other clients, and results in a reduction in degradation of these substrates (Park et al., 2013). Transport of un- or mis-folded substrates to a (juxta)nucleolar localization seems to be a more general feature for both nuclear (Nollen et al., 2001) and cytosolic proteins (Miller et al., 2015). Targeting of substrates to these structures depends on, again, Sis1 and the v-snare protein Btn2 (Malinovska et al., 2012; Miller et al., 2015). Whether the nucleolar accumulation *per se* determines the fate of the client is, however, unlikely as the accumulation in the nucleolus also occurs to nuclear clients that require refolding after heat stress (Nollen et al., 2001). Here, the accumulation of substrates in the nucleolus is reversible and accompanied by a strong sequestering of, normally cytosolic, HSPA1A (Hsp70) to the nucleolus. In line, ubiquitylation does not seem to play a role in the transfer across the nuclear envelope (Park et al., 2013; Miller et al., 2015).

What remains a question is how cytoplasmic proteins cross the nuclear barrier and whether or not posttranslational modifications play a role in this cytoplasmic-nuclear shuttling. Post-translational modifications as SUMOylation (Small Ubiquitin-like Modifier) and poly(ADP-ribosyl)ation have been implemented in protein stress responses (Haince et al., 2006; Golebiowski et al., 2009), the formation of stress bodies (Nagai et al., 2011) and also in nucleolar integrity (Finkbeiner et al., 2011; Boamah et al., 2012).

Why the nucleolus would serve as storage for proteins before folding or degradation has still remained enigmatic. Even more so, one may wonder why cytosolic substrates are translocated to the nucleus/nucleoli for degradation, given that in the cytosol proteasomes are fully active and autophagy is present. Notably, in all of these experiments the substrates used are engineered proteasomal clients. The transfer of these clients across the nuclear pore could therefore be an extension of the aforementioned ribosomal quality control mechanism, which indeed involves Sis1, and that shuttling for these substrates may occur co-translationally. However, adding an nuclear localization signal increases the degradation speed significantly (whereas adding a NES inhibits it) (Park et al., 2013), suggesting that the nuclear shuttling of cytoplasmic substrates is not that efficient and probably not coupled to translation.

One could hypothesize that substrates are normally already shuttling between the cytosol and nucleus and that degradation is simply faster in the nucleus in which the proteasomal concentration is higher (Russell et al., 1999). However, this would suggest that the capacity of the UPS in the cytoplasm is rate limiting, which seems not to be the case (Park et al., 2013). So, shuttling of un- or mis-folded clients to the nuclear/nucleolar compartment indeed seems an active, regulated process. Perhaps the cytosolic compartment, or the organelles herein, are more vulnerable to proteotoxic stress and the re-localization to the nucleus/nucleolus serves to avoid immediate cellular arrest or death. However, given the complex processes that take place in the nucleus, and the presence of the genetically vulnerable rDNA in the nucleolus, this might hamper nuclear processes such as transcription and replication.

## CFTR; escaping initial quality control is not enough

One substrate that illustrates the complexity of quality control is the disease-associated form of cystic fibrosis transmembrane conductance regulator (CFTR) (Younger et al., 2006; Okiyoneda et al., 2010). For some of the CFTR mutations, in particular the deletion of phenylalanine at position 508 (CFTR^delta508^) (Lukacs and Verkman, 2012), premature degradation is thought to underlay CF-pathology. This deletion destabilizes or kinetically traps CFTR, which targets this protein to the ERAD pathway. Numerous chaperones, including DNAJs and HSPAs, and E3 ligases have been implicated in the clearance of mutant CFTR. CFTR^delta508^ is ubiquitylated either by the Hrd1 E3 ligase complex or the Rma1/Derlin1 E3 ligase complex and the GP78 E4 ligase in a process that is enabled by the membrane embedded DNAJB12, after which retro-translocation by p97 and degradation occurs (Younger et al., 2006; Grove et al., 2011).

Skipping this initial ERAD quality control enables part of the protein to arrive at the plasma membrane to fulfill its function (Kälin et al., 1999). Unfortunately, this rescued mutant protein is still degraded, now by a different set of chaperones and E3 ligases, involving DNAJA1, DNAJB2, HSPA8, HSPA1A, Bag1, and the E3 ligase CHIP (C-terminal Hsc70/HSPA8 interacting protein) (Meacham et al., 2001; Okiyoneda et al., 2010). CHIP has a well-known function in cytoplasmic protein quality control, it binds directly to HSPA and is involved in ubiquitylating HSPA clients that need to be degraded. The work on CFTR^delta508^ nicely illustrates that each cellular compartment has a different set of protein quality control components. Moreover, it points out that in the lifetime of a protein it encounters various and different quality checks.

## Summary

The examples of substrates in the ERAD pathway and protein quality control during translation illustrated that for certain substrates a molecular chaperone (a DNAJ) is required for ubiquitylation while this is unclear for others.

The “holdase” function of DNAJs is well suited to assist E3 ligase function for those substrates that are intrinsically unstable and thus would misfold, form aggregates or fibrils in the absence of a molecular chaperone. Intriguingly, for some of these substrates HSPAs are necessary for degradation but not for ubiquitylation, indicating that they act after the E3 ligase but before proteolysis. Perhaps to prevent aggregation in the brief moment a substrate is delivered to the proteasome. Instead, the energy provided by the ATP hydroslysis of Hsp70s could also facilitate the release of the DNAJ and the E3 ligase from the ubiquitylated substrate (Figure 1B right panel). This un-entanglement-function of HSPA would not be required for substrates that come from the ER lumen, as both E3 ligase and DNAJ are embedded in the membrane, and retro-translocation is sufficient to clear the substrate from these factors. The view that degradation substrates are immediately marked for destruction without attempts to fold the substrate is not ruling out that futile folding can lead to degradation as well (Figure 1A left panel), but indicates that the degradation pathways are even more intertwined with the folding machinery as was previously thought. In that regard, it is not unlikely that the HSPA cycle, besides its canonical role in protein folding, could function to untangle the substrate-E3/E2 ligase complexes for transfer of regular enlisted clients to the proteasome as well (Mehnert et al., 2015).

### Conflict of interest statement

The authors declare that the research was conducted in the absence of any commercial or financial relationships that could be construed as a potential conflict of interest.
